# Factors That Impact Evaluation of Left Ventricular Systolic Parameters in Myocardial Perfusion Gated SPECT with 16 Frame and 8 Frame Acquisition Models

**DOI:** 10.4274/mirt.49368

**Published:** 2018-06-07

**Authors:** Mojtaba Ansari, Hoda Hashemi, Mehdi Soltanshahi, Mohsen Qutbi, Zahra Azizmohammadi, Faraj Tabeie, Hamid Javadi, Esmail Jafari, Maryam Barekat, Majid Assadi

**Affiliations:** 1 Department of Nuclear Medicine, Imam Hossein Hospital, Shahid Beheshti University of Medical Sciences, Tehran, Iran; 2 Department of Nuclear Medicine, Taleghani Educational Hospital, School of Medicine, Shahid Beheshti University of Medical Sciences, Tehran, Iran; 3 Golestan Research Center of Gastroenterology and Hepatology (GRCGH), Golestan University of Medical Sciences (GUOMS), Gorgan, Iran; 4 The Persian Gulf Nuclear Medicine Research Center, Bushehr University of Medical Sciences, Bushehr, Iran; 5 Regenerative Medicine Department, Royan Institute, Tehran, Iran

**Keywords:** Perfusion gated SPECT, 16 frames, 8 frames, systolic parameters

## Abstract

**Objective:**

Evaluating the effects of heart cavity volume, presence and absence of perfusion defect, gender and type of study (stress and rest) on the difference of systolic parameters of myocardial perfusion scan in 16 and 8 framing gated SPECT imaging.

**Methods:**

Cardiac gated SPECT in both 16 and 8 framing simultaneously and both stress and rest phases at one-day protocol was performed for 50 patients. Data have been reconstructed by filter back projection (FBP) method and left ventricular (LV) systolic parameters were calculated by using QGS software. The effect of some factors such as LV cavity volume, presence and absence of perfusion defect, gender and type of study on data difference between 8 and 16 frames were evaluated.

**Results:**

The differences in ejection fraction (EF), end-diastolic volume (EDV) and end-systolic volume (ESV) in both stress and rest were statistically significant. Difference in both framing was more in stress for EF and ESV, and was more in rest for EDV. Study type had a significant effect on differences in systolic parameters while gender had a significant effect on differences in EF and ESV in rest between both framings.

**Conclusion:**

In conclusion, results of this study revealed that difference of both 16 and 8 frames data in systolic phase were statistically significant and it seems that because of better efficiency of 16 frames, it cannot be replaced by 8 frames. Further well-designed studies are required to verify these findings.

## Abstract (tr)

**Amaç:**

Kalp boşluk hacmi, perfüzyon defekti varlığı veya yokluğu, cinsiyet ve inceleme tipinin (istirahat-stres) 16 ve 8 frame modeli ile gated SPECT görüntülemede miyokard perfüzyon sintigrafisi sistolik parametrelerindeki farklılık üzerindeki etkilerini incelemektir.

**Yöntem:**

Elli hastada eş zamanlı olarak hem 16 hem 8 frame hem stres hem dinlenme fazları ile tek-gün protokolü ile kardiyak gated SPECT uygulandı. Veriler geri filtreleme yansıması (FBP) yöntemi ile rekonstrükte edildi ve QGS yazılımı kullanılarak LV sistolik parametreleri hesaplandı. LV boşluk hacmi, perfüzyon defekti varlığı ya da yokluğu, cinsiyet ve inceleme tipi gibi bazı faktörlerin 16 ve 8 frame modeli ile gated SPECT görüntülemede miyokard perfüzyon sintigrafisi sistolik parametrelerindeki farklılık üzerindeki etkileri incelendi.

**Bulgular:**

EF, EDV ve ESV’de hem stres hem istirahattaki fark istatistik olarak anlamlı idi. Her iki frame ile EF, EDV ve ESV’de artmış fark sırasıyla stres, istirahat ve streste idi. İnceleme türünün sistolik parametreler üzerinde anlamlı etkisi vardı, benzer şekilde cinsiyetin istirahat halinde her iki bölme yöntemi arasında EF ve ESV farkı üzerinde anlamlı etkisi görüldü.

**Sonuç:**

Sonuç olarak, bu çalışma sonuçları hem 16 hem 8 frame ile elde edilen sistolik verinin istatistiksel olarak anlamlı olduğunu ortaya koymaktadır ve 16 frame modelinin daha etkin olması nedeniyle 8 frame ile değiştirilemeyeceğini öne sürmektedir. Bu bulguların ileride iyi planlanmış çalışmalar ile doğrulanması gerekmektedir.

## Introduction

Currently, gated SPECT is used for evaluation of left ventricular (LV) systolic and diastolic functions and data are acquired by the electrocardiographic signal using a specific number of interval, from R wave to next R wave (1).

Each image is generated by counts which are accumulated during each of these intervals. Each interval (cardiac cycle) can be divided into several frames. The choice of best framing interval in myocardial perfusion gated SPECT is still an unresolved issue. It is suggested that using 8-frames leads to better count density in each frame while higher framing intervals, like 16-frames, yield more accurate results (2). 

It is reported that 8-frame gated SPECT is likely to underestimate the ejection fraction (EF) as compared to other standard modalities like magnetic resonance imaging (MRI) (3,4) or equilibrium radionuclide angiography (ERNA) (5). It was stated that 8-frame gated SPECT generated smaller end-diastolic volume (EDV), larger end-systolic volume (ESV) and lower EF as compared to 16-frame gated SPECT (1,2). In a study, systolic parameters of 8, 16, 32 frames had been compared to results of ERNA. It showed that LVEF in gated SPECT underestimates as compared to ERNA and also this underestimation reduces by increasing the number of frames. This study showed that by increasing the frame, the LVEDV, LVESV, and LVEF were increased, decreased and increased, respectively (5). Vallejo et al. (6) showed that in the presence of perfusion defects, quantitative gated SPECT (QGS) overestimated the volume as compared to MRI as a standard modality.

Total perfusion deficit (TPD) is a parameter showing both the extent and severity of myocardial perfusion abnormality, and it is calculated by QGS (7). Kurisu et al. (8) used TPD as a quantitative parameter of myocardial perfusion for evaluating accuracy of QGS measurements and showed that TPD can impact systolic parameters and results in differences in ESV, EDV and EF.

The aims of this study include evaluating the effect of heart cavity volume, presence and size of perfusion defect, gender and type of study (stress and rest) on the differences in systolic parameters in myocardial perfusion scan in 8 and 16 frames gated SPECT imaging.

## Materials and Methods

This is a cross-sectional study. The patients were chosen randomly between people admitted for myocardial perfusion SPECT in Nuclear Medicine Department of a referral university affiliated hospital in Iran. The exclusion criteria included patients with severe arrhythmias (more than one ectopic beat in 6 heart cycles) (9), cardiomyopathy, a history of myocardial infarction, and patients in whom the modality is unable to determine their heart range due to abnormalities (10). 

After injection of 740 MBq of Tc-MIBI, acquisition for each patient was started after at least 60 minutes of rest and at least 30 minutes after exercise peak or pharmacologic stress.

Gated SPECT, then, was performed in both 8 and 16 frames at the same time for both rest and stress phases with a one-day protocol by using Dual head modality, high-resolution collimator and energy window of 20% for 140 KeV.

Myocardial perfusion SPECT was acquired in 64*64 matrix with 32 projections and 30 seconds for each projection.

Data were reconstructed by using filter back projection and cut off: 0.5. LVEF, LVESV and LVEDV were calculated by QGS software. V2 software was used for image reconstruction with different gating frame (10). Attenuation correction and scatter correction were not used. LVEF, LVESV and LVEDV were obtained automatically (9).

For obtaining quantitative perfusion defect as summed stress score (SSS), summed rest score (SRS) and summed difference score (SDS), polar map was used. SDS ≥2 and SDS >7 were considered as ischemia and severe ischemia, respectively (10).

LV cavity volume was obtained by ungated images and the mean volume of 8 and 16 frames in both rest and stress, separately, was considered as the heart cavity volume.

In addition, mean EDV of 8 and 16 frames in stress and rest, separately, was used as a criterion for the left ventricle volume.

After completion of sample size, the difference of systolic parameters of the two aforementioned methods and effects of size of the heart cavity, presence and size of perfusion defect, gender and type of study (stress and rest) on data difference were evaluated with SPSS (T test) and Bland-Altman analysis. P-value less than 0.05 was considered as statistically significant.

### Ethics Committee Approval and Informed Consent

This study complies with the Declaration of Helsinki, and it was approved by the Ethics Committee of Shahid Beheshti University of Medical Sciences (registration number: 75/1394-1395) and informed consent was obtained from all participants.

## Results

In 56 evaluated patients in this study, 4 patients were excluded due to inability of the modality to find the heart range, 1 patient due to silent MI and 1 patient due to arrhythmia. The study group consisted of 50 patients including 20 males and 30 females. Nine patients underwent stress with exercise test and 41 patients with pharmacologic stress. The mean age was 54.02 years with 11.11 standard deviation (SD) and a range of 30-79 years.

The mean SSS and mean SDS were calculated as 7.1 with 3.02 SD and 4.5 with 2.9 SD, respectively.

The EDV in stress phase was 79.12±30.44 for 16 frames and 78.08±29.67 for 8 frames, while this value in rest phase was determined as 82.40±30.12 for 16 frames and 81.82±29.60 for 8 frames.

The ESV in stress phase was calculated as 27.44±15.29 for 16 frames and 29±16.17 for 8 frames, and as 28.06±15.85 for 16 frames and 30.90±17.18 for 8 frames in rest phase. 

The EF in stress phase was identified as 0.67±0.07 for 16 frames and 0.63±0.08 for 8 frames, while the EF in rest phase was 0.66±0.11 for 16 frames and 0.64±0.07 for 8 frames (Table 1).

The Pearson correlation coefficient between 8 and 16 gated framings in stress and rest phase was determined as 0.957 and 0.956, respectively, and in total it was calculated to be 0.852.

**Figure 1:**
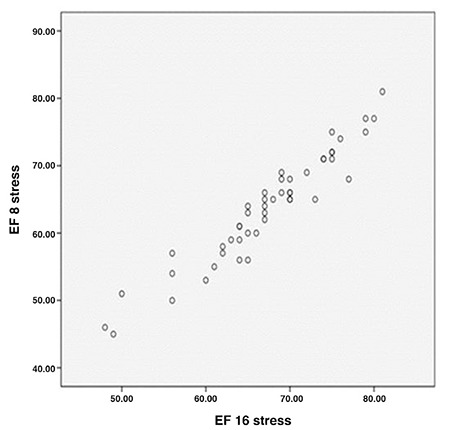
Comparison of ejection fraction between 8 and 16 frames in stress *EF: Ejection fraction*

**Figure 2:**
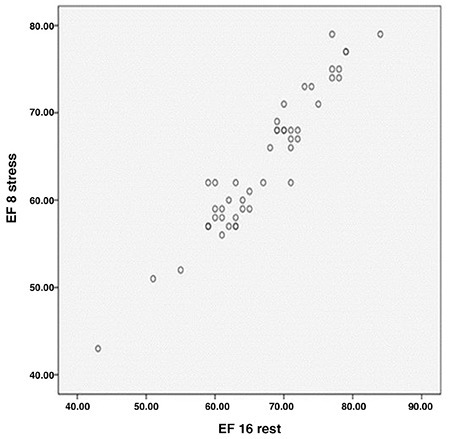
Comparison of ejection fraction between 8 and 16 frames in rest *EF: Ejection fraction*

**Figure 3:**
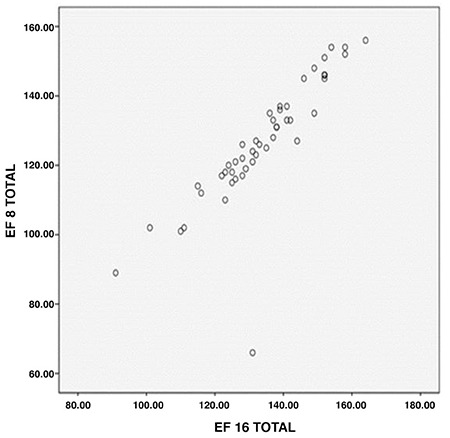
Comparison of ejection fraction between 8 and 16 frames in stress and rest *EF: Ejection fraction*

EF was higher in both stress and rest phases with both types of framing in females than males, while EDV and ESV were larger in both stress and rest phases with both types of framing in males (Figure 1, 2, 3).

EF difference between these two types of framing was obtained at 95% confidence interval of 3.05%±2.1 in stress and at 95% confidence interval of 2.92%±1.8 in rest (Figure 1, 2, 3).

95% confidence interval for difference of EDV between 8 and 16 framing was 1.73 mL±1.12 in stress and 1.98 mL±1.14 in rest. Also, 95% confidence interval for difference of ESV between 8 and 16 framing was -2.34 mL±1.9 in stress and -1.92 mL±1.6 in rest.

In the evaluation of systolic factors between 8 and 16 framings, the difference of LVEF, LVEDV, LVESV in both stress and rest phases were statistically significant (EF: p-value <0.001, ESV: p-value=0.005).

Evaluation of the effects of phase (stress and rest) on the difference of systolic parameters between two types of framing revealed that the effect of this variable on ΔEF (p-value=0.036, more difference was seen in stress), ΔEDV (p-value=0.04, more difference was seen in rest) and ΔESV (p-value=0.04, more difference was seen in stress) was statistically significant.

In evaluating effect of gender on difference in systolic parameters between 8 and 16 frames, the impact of this variable on ΔEF in rest (p-value=0.038) and on ΔESV in stress (p-value=0.030) between 8 and 16 frames was statistically significant. Mean ΔEF between 8 and 16 frames in rest phase was 3.48% for males and 2.066% for females, and this correlation for ΔESV was inverse (males<females). Effect of gender on ΔEDV was not statistically significant between 8 and 16 frames in stress (p=0.441) and in rest (p=0.083), and on ΔESV in stress (p=0.88).

With the unification of volumetric variable between both genders, there was no statistically significant in the difference of systolic parameters in both 8 and 16 frames between males and females.

Evaluation of the effects of (presence and absence of) perfusion defect on difference in systolic parameters between 8 and 16 frames showed that this variable did not have a statistically significant impact in both stress and rest on ΔEDV (stress: p=0.693; rest: p=0.78), ΔEF (stress: p=0.513; rest: p=0.964), DESV (stress: p=0.533; rest: p=0.159).

SSS and SDS had no significant effect on the related variable of systolic parameters between 8 and 16 frames.

The mean LV volume (non-gated) of 8 and 16 frames in stress had significant correlation with ΔESV in stress (Spearman correlation coefficient: 0.359, p=0.011) and rest (Spearman correlation coefficient: 0.290, p=0.041). Also, mean LV volume (non-gated) of 8 and 16 frames in rest had significant correlation with ΔESV in stress (Spearman correlation coefficient: 0.337, p=0.017) and rest (Spearman correlation coefficient: 0.333, p=0.018). The mean LV volume (non-gated) of 8 and 16 frames had no significant correlation with ΔEF and ΔEDV in both stress and rest.

The mean LVEDV of 8 and 16 frames in stress showed significant correlation with ΔESV in stress (Spearman correlation coefficient: 0.336, p=0.017) and rest (Spearman correlation coefficient: 0.324, p=0.022). However, mean LVEDV of 8 and 16 frames did not show a significant correlation with ΔEF and ΔEDV.

In our observation, 4 patients showed EF drop more than 5 percent after stress as compared to rest condition in both 8 and 16 frames imaging.

## Discussion

The results of 8 and 16 frames gated imaging in both stress and rest of 50 patients were compared in this study. The difference of EF, EDV and ESV in both stress and rest was statistically significant between 8 and 16 frames. EF in 16 frames was higher than 8 frames in both stress and rest. ΔEF in stress was more than rest in comparison of 16 frames and 8 frames. In both stress and rest, EDV was larger in 16 frames than 8 frames and ESV in 8 frames. ΔEDV in rest and ΔEF and ΔESV in stress were more in 16 frames as compared to 8 frames.

When gated SPECT was introduced, 8-interval framing has been used as the standard modality (11). Eight frames were chosen because of the need to achieve a high count in each frame for determination of cardiac walls and assessment of regional wall motion without prolonging the acquisition time (12). However, Germano et al. (9) reported that EF was underestimated by using 8 frames as compared to 16 frames, a finding that was confirmed by studies comparing EF obtained by gated SPECT with other reference standards such as ERNA and MRI (5,13,14,15).

EF in females and EDV and ESV in males were higher in both stress and rest and in both types of 8 and 16 frames imaging that is consistent with Trägårdh et al. (16) study. Nevertheless, Moslehi et al. (17) reported that the difference in systolic parameters was only observed in stress EF. Inaccurate estimates of volumes in small hearts might lead to higher EF and smaller ESV and EDV in women (18).

Since 16 frames had been introduced for obtaining diastolic factors, several studies evaluating differences in measurement of systolic factors obtained with 8 and 16 frames have been performed, with contradictory results. Only a few studies assessed the differences related directly to framing in the same patient. Navare et al. (2) have exchanged results of 16 frames to 8 frames and then by using QGS algorithms, they reported that higher LVEDV, smaller LVESV and higher LVEF in 16 frames than 8 frames and suggested to using 16 frames instead of 8 frames. These results are similar to the findings in the present study. Kumita et al. (19) had acquired 32 frames and then at each projection angle, they combined it into 16-frame and 8-frame gated data set. EDV, ESV and EF had been calculated by using QGS algorithms and had been compared with ERNA. Combining the 32-frame data into 16-frame and 8-frame data sets generated a smaller LVEDV and a larger LVESV, and LVEF was significantly lower in accordance with the decreasing number of frames. Compared with ERNA studies, the Bland-Altman method showed underestimated LVEFs and larger 95% limits of agreement in lower framing gated SPECT. Kurisu et al. (1) reported that 8 frames compared to 16 frames underestimates diastolic parameters as well as systolic parameters, and suggested that framing rate should be taken into consideration when interpreting these parameters or comparing data from different studies.

Several methods have been introduced for image reconstruction. Schaefer et al. (20) used QGS and 4D-MSPECT algorithm for comparison of 16 and 8 gated framing in 120 patients and observed smaller difference for EF with domain difference of 7% by using QGS as compared to 17% in 4D-MSPECT. Domain differences of ESV and EDV based on Bland-Altman analysis were 10 mL against 21 mL and 12 mL against 32 mL in QGS as compared to 4D-MSPECT, respectively. In addition, QGS was more accurate in determining heart range.

Montelatici et al. (10) compared 8 and 16 framing, independently, at the same time and reported underestimation of EF and EDV and overestimation of ESV in 8 frames in both stress and rest. However, the differences were very limited and smaller than that reported in other studies. Also, there was a compliance in both 8 and 16 frames for finding patients with stress-induced EF drop that indicated the lack of the superiority of one method over the other. These results are similar to the results of the present study in the way that Montelatici et al. (10) reported the mean ΔEF of 16 and 8 frames in stress as 2.8% with 95% confidence interval of 2.5-3.2 and in rest as 2.7% with 95% confidence interval of 2.3-3.1. In the present study, mean ΔEF of 16 and 8 frames in stress was calculated as 3.05% with 95% confidence interval of 1-5.1 and in rest as 2.92% with 95% confidence interval of 1.4-1.7. The higher difference in the present study as compared to the aforementioned study may be attributed to the smaller sample size.

Presence or absence of perfusion defect, SSS or SDS had no significant effect on differences of the variables related to systolic parameters between 8 and 16 frames, which are consistent with Montelatici (10) study. But Sciagrà et al. (21) confirmed that there is a significant relationship between infarct size and LV volumes and LVEF. Furthermore, in addition to infarct size, the infarct location influences LVEF and volumes, with lower LVEF values in anterior compared to lateral or inferior infarctions of the same extent, and higher EDV and ESV in anterior compared to inferior or lateral infarctions. Also, this study showed a significant correlation with LV volumes and LVEF. Vallejo et al. (6) reported that QGS overestimated the EDV in the presence and absence of perfusion defect. However, in the presence of a perfusion defect, overestimation was worse. ESV and LVEF were also overestimated in the presence of a perfusion defect.

### Study Limitations

The main limitation of the study is the lack of the assessment of clinical impact of the acquired data. In other words, it is possible that the difference between 16 and 8 frames does not have an effect on treatment approach. Another limitation was the lack of comparison with a gold standard test. Finally, it is possible that the observed difference between 16 and 8 frames does not indicate the superiority of one method over the other.

## Conclusion

In conclusion, according to our results, although the difference of systolic factors in 16 and 8 gated framings by using the similar protocol were estimated less than previous studies, the difference is still significant. Further studies are required to evaluate the effect of this difference on treatment approach.

## Figures and Tables

**Table 1:**
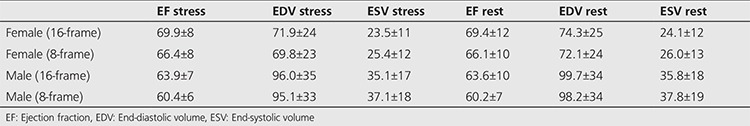
Mean values of systolic and diastolic parameters in 8 and 16 frames
